# MHCI trafficking signal‐based mRNA vaccines strengthening immune protection against RNA viruses

**DOI:** 10.1002/btm2.10709

**Published:** 2024-08-15

**Authors:** Yupei Zhang, Songhui Zhai, Shugang Qin, Yuting Chen, Kepan Chen, Zhiying Huang, Xing Lan, Yaoyao Luo, Guohong Li, Hao Li, Xi He, Meiwan Chen, Zhongwei Zhang, Xingchen Peng, Xin Jiang, Hai Huang, Xiangrong Song

**Affiliations:** ^1^ Department of Critical Care Medicine Frontiers Science Center for Disease‐related Molecular Network, State Key Laboratory of Biotherapy and Cancer Center, West China Hospital, Sichuan University Chengdu Sichuan China; ^2^ Department of Pediatrics West China Second University Hospital, Sichuan University Chengdu Sichuan China; ^3^ State Key Laboratory of Quality Research in Chinese Medicine, Institute of Chinese Medical Sciences, University of Macau Macau China

**Keywords:** framework, immune protection, MITD, mRNA vaccines, RNA virus

## Abstract

The major histocompatibility complex class I (MHCI) trafficking signal (MITD) plays a pivotal role in enhancing the efficacy of mRNA vaccines. However, there was a lack of research investigating its efficacy in enhancing immune responses to RNA virus infections. Here, we have developed an innovative strategy for the formulation of mRNA vaccines. This approach involved the integration of MITD into the mRNA sequence encoding the virus antigen. Mechanistically, MITD‐based mRNA vaccines can strengthen immune protection by mimicking the dynamic trafficking properties of MHCI molecule and thus expand the memory specific B and T cells. The model MITD‐based mRNA vaccines encoding binding receptor‐binding domain (RBD) of SARS‐CoV‐2 were indeed found to achieve protective duration, optimal storage stability, broad efficacy, and high safety.

**Translational Impact Statement**

Our findings provide a groundbreaking framework for the advancement of MITD‐based antigen design for RNA viruses. This design optimizes the efficacy of mRNA vaccines, offering substantial promise for future clinical applications.

## 1 INTRODUCTION

The inherent single‐stranded and unstable nature of RNA viruses facilitates their rapid mutation and contributes to their high infectiousness.^1^ In recent years, research on mRNA vaccines aimed at preventing RNA virus pandemics has expanded significantly, entering clinical trials for various viruses, including influenza,^2^, ^3^ rabies virus,^4^, ^5^ Zika virus,^6^, ^7^ respiratory syncytial virus (RSV),^8^ human metapneumovirus (hMPV),^9^ human immunodeficiency virus (HIV),^10^ Nipah virus (NIV),^11^ and Ebola virus.^12^ However, to date, only mRNA vaccines targeting severe acute respiratory syndrome coronavirus 2 (SARS‐CoV‐2) have been officially launched.^13^ A contributing factor to the relatively slow clinical progress of other vaccines may be that the elicitation of both humoral and cellular antigen‐specific immune responses is insufficient to facilitate virus clearance, mitigate the clinical severity of infection, and induce lasting immune memory.^14^ Notably, it has been observed that the presentation of major histocompatibility complex (MHC) epitopes is an inefficacious process.^15^ Even for high‐affinity MHC ligands, only one peptide per 10,000 degraded molecules is presented, suggesting a significant opportunity to enhance vaccine efficiency.^16^ Consequently, this realization has pivoted our focus towards optimizing the sequence design of mRNA vaccines against RNA viruses by investigating the intricacies of antigen processing and presentation.

In addition to the N‐terminal signal peptide, the transmembrane and cytosolic domains of the Major Histocompatibility Complex (MHC) class I molecule, collectively referred to as the MHC class I Trafficking Domain (MITD), have been demonstrated to regulate antigen recycling among various endosomal compartments, facilitating cross‐presentation in dendritic cells (DCs). Recent studies have incorporated MITD into tumor vaccines, resulting in enhanced antigen presentation through the efficient expansion of antigen‐specific CD4^+^ and CD8^+^ T cells.^17^, ^18^ This enhancement is attributed to improved organelle localization of the antigen.^19^, ^20^, ^21^ Notably, mRNA sequences of antigens linked with MITD at the C‐terminal have shown superior performance compared to those lacking MITD.^18^ In the context of the immune response against RNA virus infection, the processes of antigen processing and presentation to CD4^+^ and CD8^+^ cells are critical. Therefore, incorporating MITD‐based designs represents a promising new strategy in the development of highly efficacious mRNA vaccines against RNA virus infections.

**FIGURE 1:**
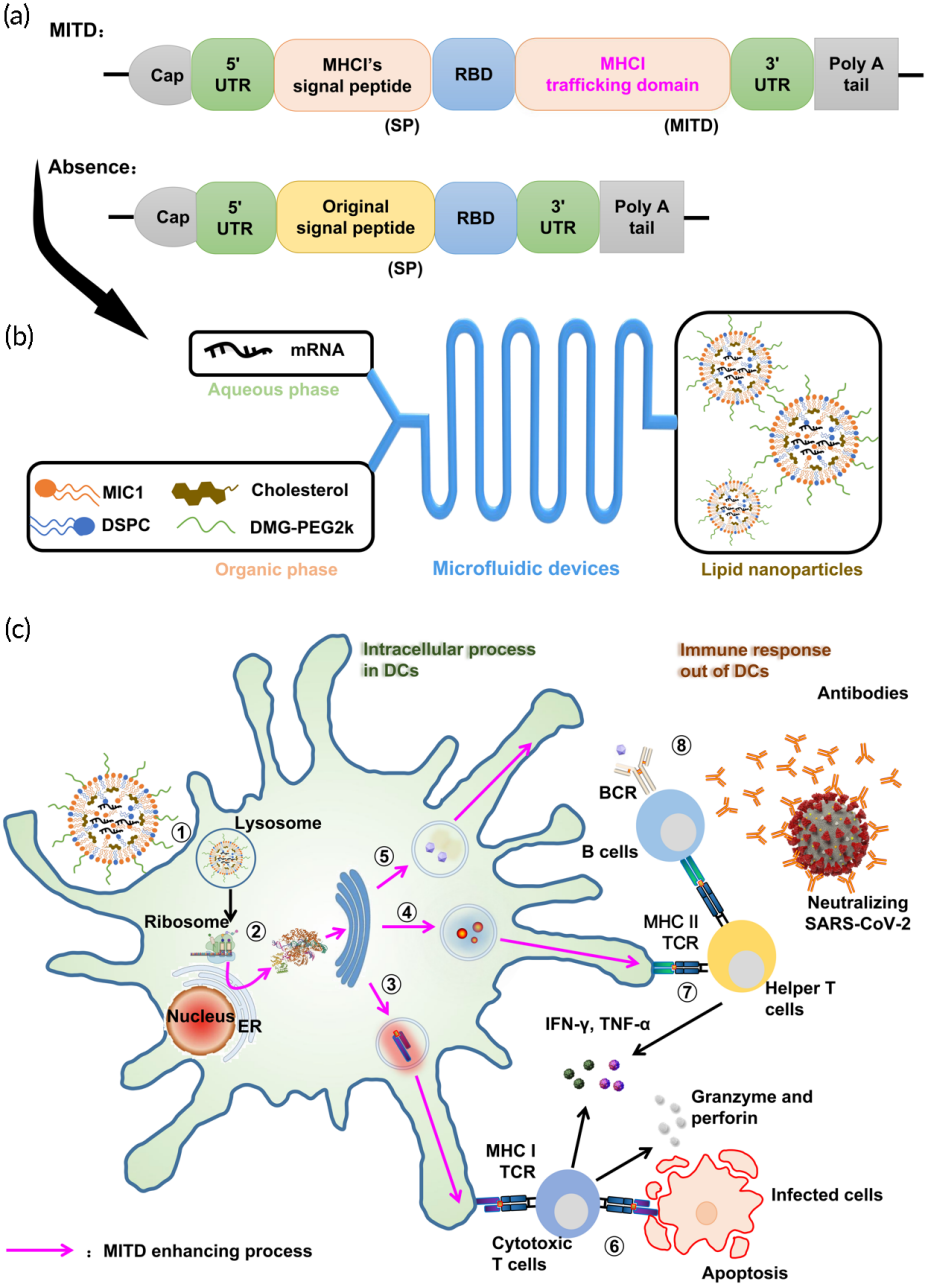
Graphical abstract of MITD‐based mRNA vaccines preventing against the RNA virus SARS‐CoV‐2 infection. (a) Design of RBD antigen mRNAs: the MITD group includes coding sequence (CDS) of mRNAs with MITD, while the Absence group comprises CDS of mRNAs without MITD. (b) Preparation of mRNA vaccines: vaccines are formulated using a microfluidic device. (c) Mechanism: ① DCs endocytose the mRNA vaccines encoding the antigen. ② The mRNAs evade lysosomal degradation and are translated in the cytoplasm. Newly synthesized peptide chains pass through the endoplasmic reticulum (ER) membrane, undergoing processing and modification in the ER and Golgi apparatus. MITD sequences guide these peptides to specific cellular compartments, such as the ER, Golgi apparatus, endosomes, and plasma membrane. This enhances the presentation of ③ cytotoxic T lymphocyte epitopes via MHC I and ④ helper T lymphocyte epitopes via MHC II. Additionally, ⑤ linear B lymphocyte epitopes are secreted. ⑥ Activated cytotoxic T cells release perforin and granzyme, and induce apoptosis in infected cells. ⑦ Helper T cells are also activated. ⑧ B cells, upon receiving antigenic signals through BCRs and secondary signals from activated helper T cells via CD40, differentiate into plasma cells. These plasma cells produce antibodies that neutralize SARS‐CoV‐2.

To test the aforementioned hypothesis, the recent pandemic of Coronavirus Disease 2019 (COVID‐19), caused by SARS‐CoV‐2, was selected as a model for RNA virus infection. Here, MITD‐based binding receptor‐binding domain (RBD) mRNA vaccines were designed and developed. RBD^WT^ mRNAs vaccines containing MITD and lack of MITD in our study were tested to compare their RBD expression level in vitro, and their immune response of antigen specific memory B and T cells against the SARS‐CoV‐2 in vivo. The process undertaken in this research is illustrated in Figure 1. Intriguingly, we observed that the inclusion of MITD sequences in mRNA vaccines elicited a more potent antigen‐specific immune response compared to their MITD‐lacking counterparts, even at equivalent levels of antigen expression. Furthermore, we assessed the temporal stability of the protection and the storage stability of these vaccines against the later‐emerging Omicron BQ.1 variant antigen. Our findings lay the groundwork for the continued development of MITD‐based antigen design, aimed at enhancing the efficacy of mRNA vaccines in combating the pandemic of RNA virus infectious diseases.

## 2 MATERIALS AND METHODS

### 2.1 In vitro transcription of mRNA

mRNA was transcribed in vitro utilizing T7 RNA polymerase (Vazyme Biotech Co., Ltd.), following a process of transcription from a linearized DNA template. This template comprised the optimal codons for the wild type (WT), and BQ.1 RBD from the S protein of SARS‐CoV‐2 (GenBank: P0DTC2). The signal peptide sequence originated from SARS‐CoV‐2 (GenBank: P0DTC2), while the MITD sequence with the signal peptide was derived from the major histocompatibility complex class I molecule H‐2D^b^ ^22^, ^23^ (GenBank: P01899).

### 2.2 Preparation and characterization of mRNA vaccines

The synthesis of MIC1 lipids was based on methods previously established by our group.^24^ mRNA vaccine lipid nanoparticles (LNPs) encapsulating the mRNAs were prepared using the INano L microfluidic device (Micro&Nano Biologics). The mass ratio of MIC1 to mRNA was set at 15:1. Initially, the ionizable lipid MIC1, 1,2‐distearoyl‐sn‐glycero‐3‐phosphocholine (DSPC), cholesterol, and 1,2‐dimyristoyl‐rac‐glycero‐3‐methoxypolyethyleneglycol‐2000 (DMG‐PEG2k) were dissolved in ethanol at a molar ratio of 35:16:46.5:2.5, constituting the organic phase.^24^, ^25^ Concurrently, mRNA was dissolved in a citric acid buffer (pH 6.0) to form the aqueous phase. The mRNA vaccines were then produced by combining these phases in a 1:3 (v/v) ratio using the microfluidic device, at a flow rate of 12 mL/min. Subsequently, the ethanol was removed from the mRNA vaccines through ultrafiltration.

The morphology of the mRNA vaccines was visualized using 2% phosphotungstic acid staining, observed under a TEM FEI Talos F200XG2 AEMC (Thermo Fisher). The particle size and zeta potential of the mRNA vaccine LNPs were measured using a Zetasizer Nano ZS90 (Malvern). The free mRNA content (mRNA_free_) in the mRNA vaccine LNPs was quantified using the Quant‐iT RiboGreen RNA assay kit (Invitrogen). The total mRNA (mRNA_total_) was assayed with a Microfluidic micro concentration particle size analyzer (Stunner). The encapsulation efficiency was calculated as (1 − mRNA_free_/mRNA_total_) × 100%.^26^, ^27^

### 2.3 Western blotting analysis

Two hundred and ninety‐three T cells were seeded into 6‐well plates at a density of 5 × 10^5^ cells per well and incubated for 24 h. Post incubation, these cells were treated with 1 μg of mRNA vaccines, which contained the sequences of Absence and MITD. For the detection of RBD expression and the internal reference, primary antibodies anti‐SARS‐CoV‐2 RBD (1:1000, Sino Biological Inc.) and anti‐GAPDH (1:2000, HUABIO, Catalog # EM1101) were employed in the western blotting analysis. The procedures were executed in accordance with established standard methods.

### 2.4 Animal treatment

Animals, procured from SPF (Beijing) Biotechnology Co., Ltd., were housed under conditions of a 12‐h light/dark cycle at a controlled temperature, with unrestricted access to sterilized food and water. The animal study schedule was inspected by the Experimental Animal Ethics Committee of West China Hospital, and complied with the relevant regulations of national experimental animal welfare ethics with the approval number of 20221110003.

Male BALB/c mice, weighing between 18 and 20 g, were randomly assigned into six groups, with either four or six mice per group. They underwent two intramuscular immunizations with low (0.2 μg), medium (1 μg), or high (5 μg) doses of mRNA vaccines on days 0 and 14. A vehicle solution was administered in the same volume to serve as the normal control (NC). Serum samples were collected on days 14, 28, 42, and 84. Further, on day 84, certain tissues were harvested to investigate the B and T cell responses and the safety of the preparations. In a separate experiment, animals were immunized with a 5 μg dose of the mRNA vaccines on days 0 and 14. Serum samples in this study were collected on days 14, 28, 42, 56, 70, and 84. Additionally, when using preparations stored at 4°C for 14 and 28 days, serum samples were collected on day 28 following the initial vaccination.

### 2.5 Enzyme‐linked immunosorbent assay

The enzyme‐linked immunosorbent assay (ELISA) test was conducted to determine the binding titers of the sample serum. High‐binding polystyrene plates (Corning) were coated with SARS‐CoV‐2 S protein RBD from various strains, including Wildtype 2019‐nCoV (WT), B.1.617.2 (Delta), and Omicron B.1.1.529, each tagged with His‐Tag (Novoprotein, Shanghai, China) at a concentration of 1 μg/mL. The plates were incubated overnight at 4°C. Subsequent to this incubation, the plates underwent a four‐time wash with washing buffer, followed by a blocking process using 2% Bovine Serum Albumin (BSA) for 4 h at 25°C. Post‐blocking, the plates were washed twice and then incubated with serum samples. These samples, initially inactivated at 56°C for 30 min and starting at a 200‐fold dilution, were serially diluted in a twofold manner and incubated overnight at 4°C. Following another series of four washes, HRP‐conjugated anti‐mouse IgG (1:50,000, Cell Signaling Technology) was added and incubated for 2 h at 25°C. After a final set of four washes, 3,3′,5,5′‐tetramethylbenzidine (TMB, Solarbio) was applied. The enzymatic reaction was halted 30 min later with 2 mol/L sulfuric acid, and the absorbance was recorded at 450 nm using a microplate reader (Perkin Elmer). Endpoint titers were determined as the serum dilution fold exceeding control values.

### 2.6 Pseudovirus neutralization assay

Neutralization assays were performed using pseudoviruses representative of various SARS‐CoV‐2 strains, including WT, Delta, and several Omicron subvariants (B.1.1.529, BQ.1, BF.7, BA.4/BA.5, XBB.1.5, and CH.1.1), each coupled with GFP‐Luciferase (Genomeditech). Pseudoviruses were diluted in DMEM (supplemented with 10% FBS and 1% penicillin–streptomycin, Gibco) to a final concentration of 1 × 10^5^ TU/mL. Serum samples, initially diluted 90‐fold, were serially diluted threefold and incubated for 1 h at 37°C. HEK293T‐hACE2 cells (3 × 10^4^ cells) were then added to each well. Following a 48‐h incubation period, an enzyme substrate was introduced for luciferase activity detection using a microplate reader. The neutralization endpoint, NT50, was defined as the serum dilution required for 50% inhibition of luciferase activity relative to virus control samples.

### 2.7 B cell flow cytometry

The kinetics of S‐specific memory B cell responses were investigated as outlined in previous studies.^28^, ^29^ In brief, splenocytes were freshly extracted from the negative control (NC) group and five groups immunized with mRNA vaccines at day 84 post‐vaccination. The cells were first labeled with AVI‐tagged S protein (Vazyme Biotech Co., Ltd.) for 30 min at 4°C. After triple washing with PBS, the cells were stained with a panel of antibodies: FITC anti‐mouse CD16, PE anti‐mouse CD4, PE/Cyanine5 streptavidin (binding to AVI tag), PerCP/Cyanine5.5 anti‐mouse IgM, PE/Cyanine7 anti‐mouse CD20, APC/Cyanine7 anti‐mouse CD14, Pacific Blue anti‐mouse CD19, Qdot655 anti‐mouse IgD, Qdot705 anti‐mouse CD3 (all from Biolegend), and Fixable Yellow Dead Cell Stain Kit (Pacific Orange, Thermo Fisher). Following another triple wash with PBS, flow cytometric analysis was performed using a NovoCyte™ (Eisen) instrument.

### 2.8 T cell flow cytometry

Antigen‐specific memory CD4^+^ T and CD8^+^ T immune responses were further determined by ICS assay.^24^, ^26^, ^30^, ^31^, ^32^ In short, freshly extracted splenocytes obtained from NC, the mRNA vaccines immunized groups at day 84, and the S protein peptide pools of SARS‐CoV‐2 (2 μg/mL of individual peptide) were added to 24‐well plates (2 × 10^6^ cells/well) simultaneously (RBD antigen is a part of S protein in SARS‐CoV‐2). After 2 h, monensin (YEASEN, Cat: 50501ES03) was added to each well to inhibit extracellular cytokine secretion. The cells were harvested 12 h later and stained with PE anti‐mouse CD4, Percp anti‐mouse CD8, Alexa Fluor 700 anti‐mouse MHCII, BV421 anti‐mouse B220, BV510 anti‐mouse CD44, BV711 anti‐mouse CD3 (Biolegend), and Fixable Yellow Dead Cell Stain Kit (Pacific Orange, Thermo Fisher) for 40 min. Afterwards, the cells were fixed with fixation buffer for 20 min. Finally, the cells were permeabilized in 1× permeabilizing buffer (Biolegend) and stained with FITC anti‐mouse IFN‐γ, PE/Cyanine7 anti‐mouse TNF‐α (Bioss), and APC anti‐mouse IL‐4 (Biolegend). After washing three times with PBS, the cells were analyzed by flow cytometry using NovoCyte™ (Eisen).

### 2.9 Enzyme‐linked immunospot

The IFN‐γ‐based enzyme‐linked immunospot (ELISpot) assay was utilized to assess antigen‐specific T cell responses using the Mouse IFN‐γ ELISPOTPLUS kit (Mabtech), following the manufacturer's protocol. The assay plates were initially washed four times with sterile PBS (200 μL/well) and subsequently blocked with DMEM containing 10% FBS (200 μL/well, Gibco) for a minimum of 30 min at room temperature. Freshly isolated splenocytes (5 × 10^5^ cells/well) from mice vaccinated 84 days post the initial immunization, along with S protein peptide pools of SARS‐CoV‐2 (2 μg/mL per peptide), were added to the plates. The plates were incubated at 37°C in a 5% CO_2_ environment for 36 h. Following incubation, the cells were discarded, and the plates were washed five times with PBS (200 μL/well). The assay continued with the sequential addition of biotinylated IFN‐γ detection antibody, streptavidin‐ALP conjugate, and substrate, adhering to the kit instructions. Spot development was halted upon distinct appearance by washing the plates with deionized water. Spot quantification was conducted using an automatic ELISpot reader.

### 2.10 Safety evaluation

The safety of the mRNA vaccines was assessed through blood biochemical analysis of serum samples collected on day 84. Key biochemical markers, including alanine aminotransferase (ALT), aspartate aminotransferase (AST) for liver function, albumin (ALB), creatinine (CRE) and UREA for kidney function, and Creatine kinase isoenzymes (CKMB) for heart function were quantified using an automatic hematological biochemical analyzer (Hitachi). Additionally, organ samples from each mouse were processed for Hematoxylin and Eosin (H&E) staining. The stained tissues were examined, and images were captured using an Olympus‐BX 43 fluorescence microscope (Olympus).

### 2.11 Quantification and statistical analysis

Statistical analyses were conducted using a one‐way analysis of variance (ANOVA). The results were presented as mean ± standard error of the mean (SEM). A *p*‐value of less than 0.05 was considered statistically significant. All statistical computations were performed using SPSS software, version 26.0.

## 3 RESULTS

### 3.1 Preparation and characterization of mRNA vaccines

**FIGURE 2:**
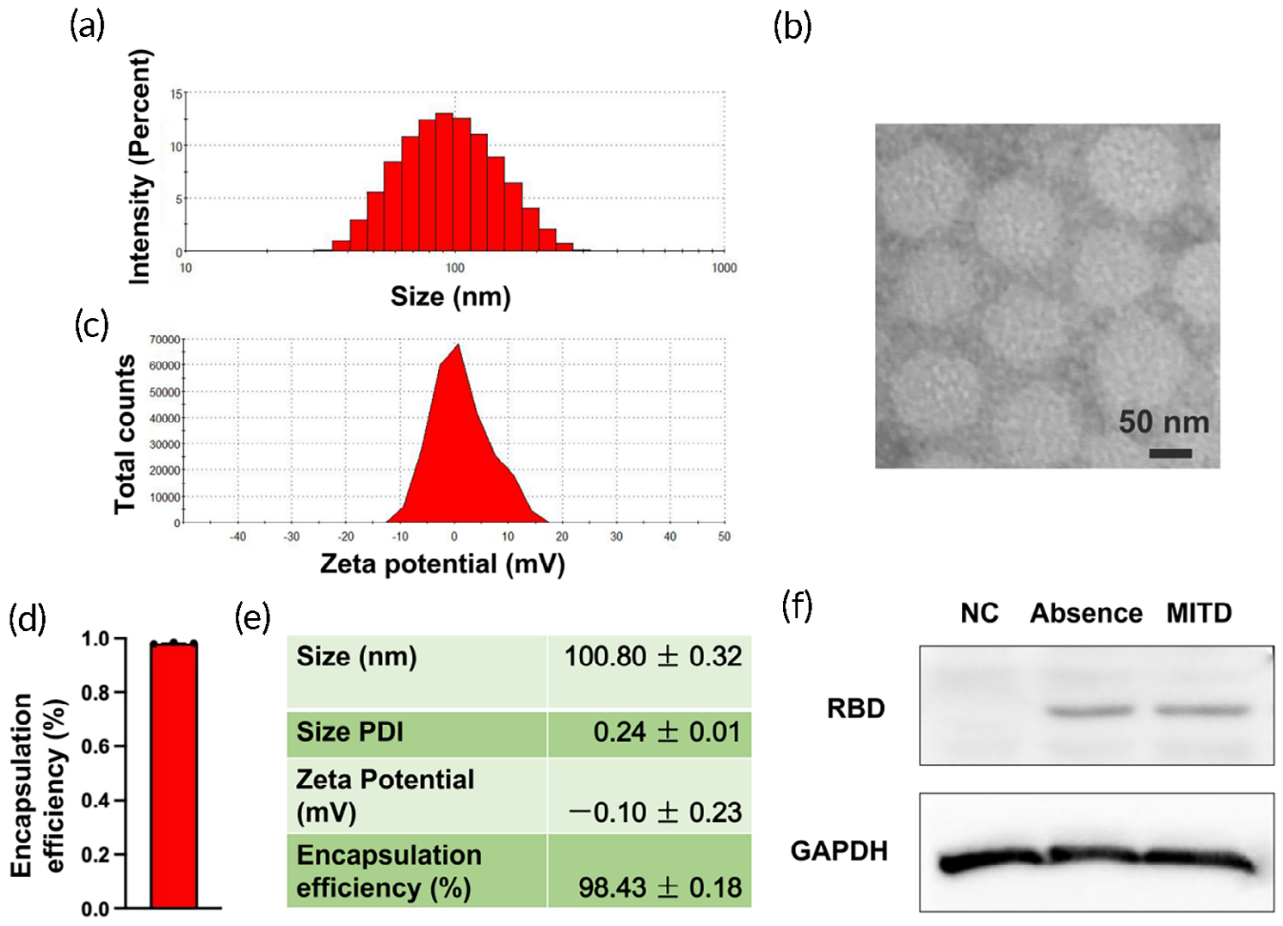
Characterization of mRNA vaccines. (a) Size distribution: this panel presents a representative size distribution graph of the mRNA vaccines. (b) Transmission electron microscopy (TEM) image: the photo shows a TEM image of mRNA vaccine, with a scale bar representing 100 nm. (c) Zeta potential distribution: this panel illustrates a representative zeta potential graph of the mRNA vaccines. (d) Encapsulation efficiency: this section quantifies the encapsulation efficiency of the mRNA vaccines. (e) Aggregate vaccine characteristics: displayed here are the size, polydispersity index (PDI), zeta potential, and encapsulation efficiency of the mRNA vaccines, with data from three independent experiments (*n* = 3). The values are expressed as mean ± SEM. (f) Intracellular and extracellular RBD expression: this graph quantifies the RBD expression levels within cells and in the culture supernatant of 293T cells transfected with the mRNA sequences illustrated in Figure 3a. The groups compared include a normal control (NC), mRNAs containing the MITD (MITD), and mRNAs in the absence of MITD (Absence). Data are presented as mean ± SEM.

**FIGURE 3:**
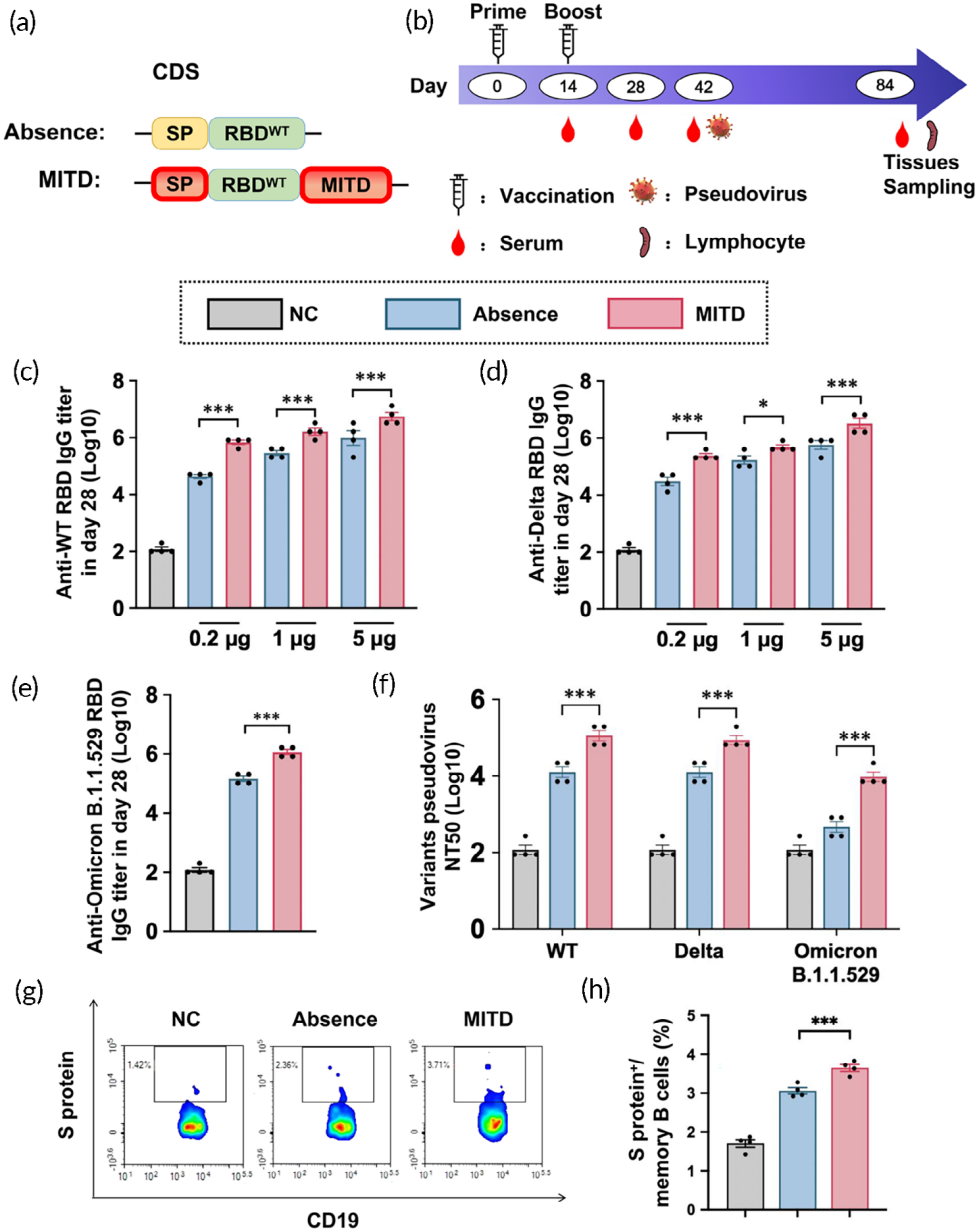
Evaluation of specific antibodies and memory B cell responses induced by MITD‐enhanced RBD^WT^ mRNA vaccines against SARS‐CoV‐2 variants. The groups compared include a normal control (NC), mRNAs containing the MITD (MITD), and mRNAs in the absence of MITD (Absence). (a) Composition of two mRNA sequences: this panel details the components of the two mRNA sequences included in the mRNA vaccines. (b) Experimental setup: the schematic outlines the experimental protocol applied to BALB/c mice. (c, d) Serum ELISA results: these panels display the ELISA results for RBD^WT^ and RBD^Delta^‐specific IgG titers at day 28 post‐vaccination in BALB/c mice, administered with 0.2, 1, and 5 μg doses of RBDWT mRNA vaccines containing MITD and lacking MITD. (e) Serum ELISA for RBD^Omicron^‐specific IgG: this graph shows the IgG titers against the RBD^Omicron^ variant at day 28 in mice vaccinated with 5 μg doses of RBD^WT^ mRNA vaccines containing MITD and lacking MITD. (f) Pseudovirus neutralization assays: presented here are the results of neutralization tests against WT, Delta, and Omicron B.1.1.529 pseudoviruses in mice, conducted at day 42 post‐administration of 5 μg doses of RBD^WT^ mRNA vaccines containing MITD and lacking MITD. (g) Flow cytometry analysis: this panel includes representative flow cytometry diagrams, and (h) quantification of S protein‐specific memory B cells: The graph quantifies S protein‐specific memory B cells at day 84 post‐vaccination. Gating strategy of flow cytometry is shown in Figure S4. The data are shown as mean ± SEM, *n* = 4. *p < 0.05, ***p < 0.001.

A microfluidic device was employed to synthesize mRNA vaccines. These vaccines comprised cholesterol for stabilization, natural phospholipids DSPC to support lipid bilayer structure, PEG derivatives to minimize aggregation and nonspecific uptake, and ionizable lipids MIC1, which bind negatively charged mRNA and enhance endosomal escape,^25^, ^33^ along with mRNAs (as illustrated in Figure 1b). Notably, in our vaccine formulation, ionizable lipid MIC1 demonstrated superior performance in terms of expression and antibody titers compared to SM‐102, a compound invented by our group and already extensively utilized in mRNA vaccines.^24^ The morphological properties of these mRNA vaccines can be seen in Figure 2b. The average size was measured at 100.8 nm, with a PDI of 0.24, a zeta potential of −0.1 mV, and an encapsulation efficiency of 98.43%, indicating stable characteristics (Figure 2a,c–e). Furthermore, the translation levels of MITD‐based RBD mRNAs in 293T cells were evaluated using Western blotting. The design sequences of the RBD^WT^ mRNA vaccines are displayed in Figure 3a, differentiating between mRNAs with MITD (CDS containing MITD) and those without (CDS in the absence of MITD). Our results indicated that adding MITD will not affect the expression levels of RBD in 293 T cells (Figure 2f).

### 3.2 MITD‐based mRNA vaccines induced stronger antibody titers and specific memory B cells against virus

Subsequently, the immunostimulatory effect of the vaccines in vivo was evaluated. BALB/c mice were intramuscularly injected with 0.2, 1, and 5 μg of both MITD and Absence‐based mRNA vaccines in their legs on days 0 and 14. Samples were collected on days 14, 28, 42, and 84, as depicted in Figure 3b. The results indicated that the MITD group exhibited higher anti‐WT and Delta RBD IgG titers on days 14 and 28, with these effects being dose‐dependent (Figures S1 and 3c,d). Similarly, the trend in anti‐Omicron B.1.1.529 RBD IgG titers on days 14 and 28 paralleled that observed for WT and Delta (Figures S2 and 3e). The persistence of the immune effect was monitored on days 42 and 84, showing a decrease in IgG titers against all three SARS‐CoV‐2 strains to some degree, yet the overall trend remained unchanged (Figure S3). Concurrently, neutralizing antibody titers against the three strains were assessed on day 42, revealing consistent findings (Figure 3f).

Memory B cells play a crucial role in combatting viral infections. A gating strategy of CD3^−^ CD4^−^ CD14^−^ CD16^−^ CD19^+^ CD20^+^ IgM^−^IgD^−^S protein^+^ was utilized to identify S protein‐specific memory B cells (Figure S4). Notably, the S protein‐specific memory B cells in the MITD group were more prevalent than those in the Absence group on day 42 (Figure 3g,h).

These findings demonstrate that the MITD‐based RBD^WT^ mRNA vaccines possess broad‐spectrum efficacy and elicit superior antibody titers compared to vaccines lacking MITD sequences.

### 3.3 MITD‐based mRNA vaccines induced stronger response of T cells against virus

**FIGURE 4:**
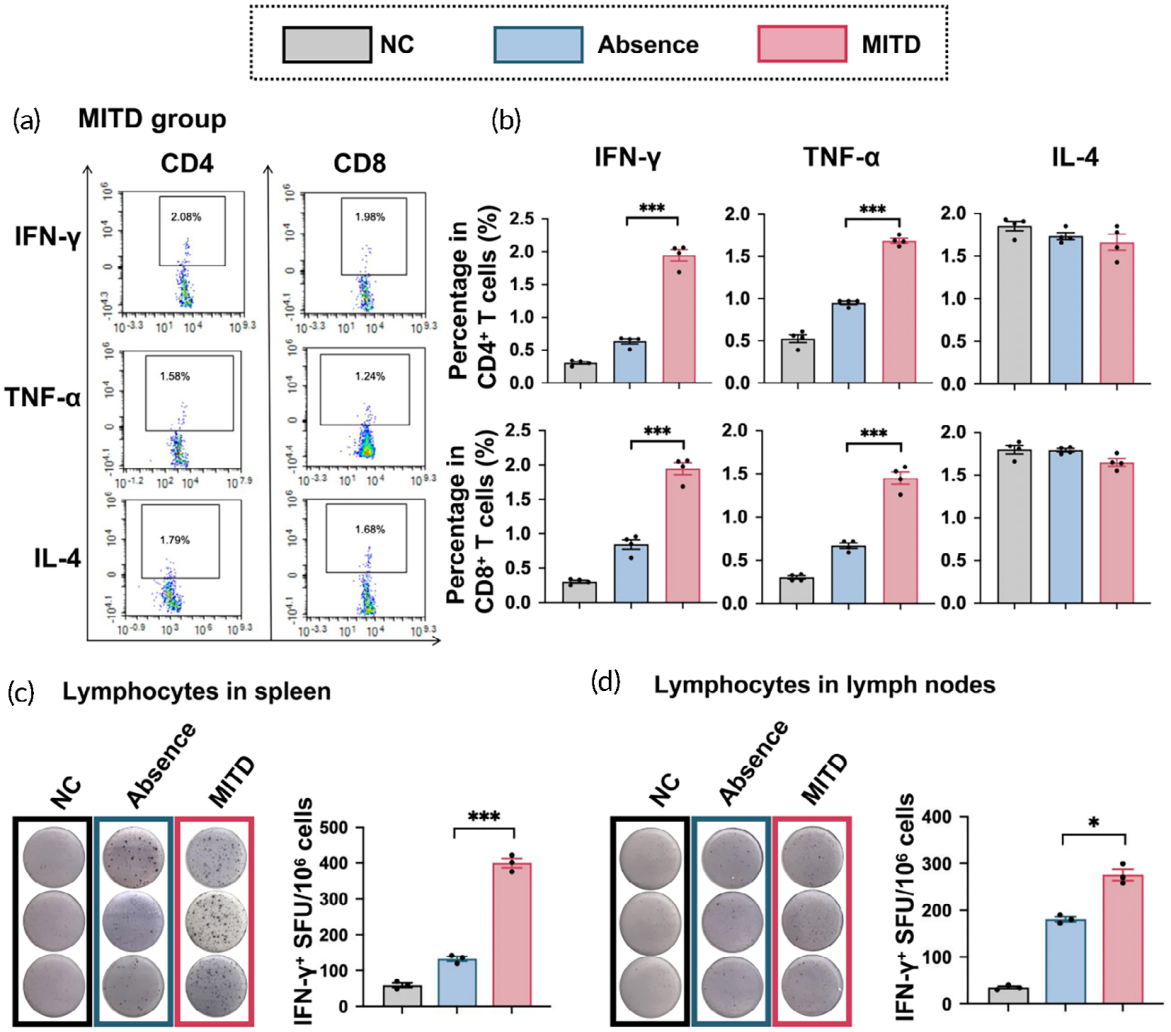
Enhancement of T‐cell response by MITD‐enhanced RBD^WT^ mRNA vaccines. At day 84, lymphocytes from spleen and lymph nodes were harvested from mice that received an intramuscular injection of 5 μg of RBD^WT^ mRNA vaccines. These lymphocytes were then exposed to peptide pools of the SARS‐CoV‐2S protein for stimulation. The groups compared include a normal control (NC), mRNAs containing the MITD (MITD), and mRNAs in the absence of MITD (Absence). Panels A and B display representative flow cytometry diagrams alongside quantifications of intracellular cytokine staining (IFN‐γ, TNF‐α, and IL‐4) in CD4^+^ and CD8^+^ memory T cells within the spleens (*n* = 4). Gating strategy of flow cytometry is shown in Figure S5. (c, d) Images and spots count quantification of ELISpot testing for IFN‐γ^+^ T cells in spleens and lymph nodes (*n* = 3). The data are shown as mean ± SEM. *p < 0.05, ***p < 0.001.

Helper T (Th) cells facilitate B cell responses, while cytotoxic T (Tc) cells directly target virus‐infected cells. Our initial step involved performing intracellular staining (ICS) on splenocytes. Flow cytometry was employed to detect IFN‐γ and Interleukin (IL)‐2 secreting memory Th1 cells, along with IL‐4 secreting memory Th2 cells, within the CD44^high^ CD4^+^ cell population. Concurrently, we also detected IFN‐γ and IL‐2 secreting memory Tc1 cells, and IL‐4 secreting memory Tc2 cells in the CD44^high^ CD8^+^ cell subset (Figures S5 and 4a). Our findings revealed that, on day 42, memory Th1 and Tc1 cell levels in the MITD group surpassed those in the Absence group. However, memory Th2 and Tc2 cell counts did not exhibit significant differences (Figure 4b).

Subsequently, ELISpot assay was performed to assess the capability of lymphocytes in the spleen and lymph nodes to secrete IFN‐γ on day 42. The representative images and statistical histograms indicated that the MITD group had a higher number of lymphocytes secreting IFN‐γ compared to the Absence group, both in the spleen (Figure 4c) and lymph nodes (Figure 4d). These results aligned with the aforementioned findings, demonstrating MITD‐based mRNA vaccines elicited a robust T cell immune response.

### 3.4 MITD‐based mRNA vaccines initiated a sustained and vigorous immune response against mutants of virus with good storage stability

**FIGURE 5:**
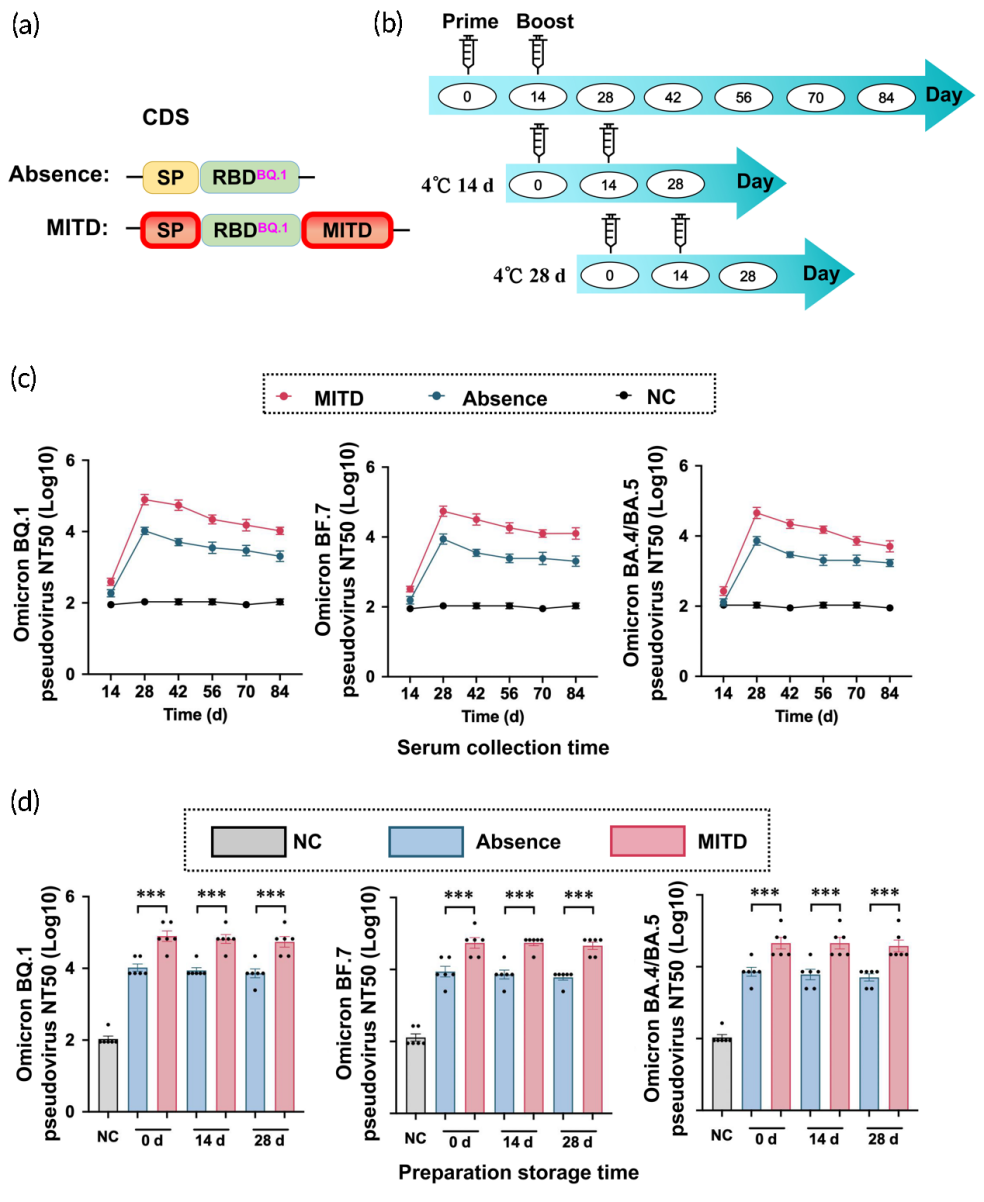
Evaluation of the immune response to MITD‐enhanced RBD^BQ.1^ mRNA vaccines against SARS‐CoV‐2 Omicron variants BQ.1, BF.7, and BA.4/BA.5. (a) Details of the mRNA sequences incorporated into the vaccines. (b) Schematic representation of the experimental setup involving BALB/c mice. We evaluated the neutralizing efficacy of serum from mice inoculated with the MITD‐based RBD^BQ.1^ and Absence mRNA vaccines against the three Omicron subvariants. The neutralization potential of serum obtained at (c) various collection times and (d) different vaccine storage durations at 4°C were assessed. The groups compared include a normal control (NC), mRNAs containing the MITD (MITD), and mRNAs in the absence of MITD (Absence). The data are shown as mean ± SEM, *n* = 6. ***p < 0.001.

Although the WT SARS‐CoV‐2, selected as the antigen, remains effective against virus variants, there has been a noted decrease in protection efficiency.^34^ Consequently, recently emerged mutant strains were incorporated as antigens to enhance protective efficacy. Following the preparation of RBD^BQ.1^ mRNA vaccines incorporating MITD and without, we assessed and compared their titers over a series of time points, and efficacy over a storage period of 0, 14, and 28 days (Figure 5a,b). Our observations revealed that over an 84‐day period, the titer stability against recent SARS‐CoV‐2 mutants, including BQ.1, BF.7, and BA.4/BA.5, was relatively consistent, maintaining stable efficacy (Figure 5c). Additionally, the vaccines were stored at 4°C for 0, 14, and 28 days before administering two doses to mice. Serum collected on day 28 post‐first vaccination was analyzed for neutralizing antibody titers against recent SARS‐CoV‐2 mutants, including BQ.1, BF.7, BA.4/BA.5, B.1.1.529, XBB.1.5, and CH.1.1. The results indicated that the mRNA vaccines with MITD exhibited ideal storage stability (Figures 5d and S6). In summary, the MITD‐based RBD^BQ.1^ mRNA vaccines demonstrated temporal stability of protection, favorable storage stability, and relatively broad‐spectrum efficacy.

### 3.5 Safety profiles of MITD‐based mRNA vaccines

**FIGURE 6:**
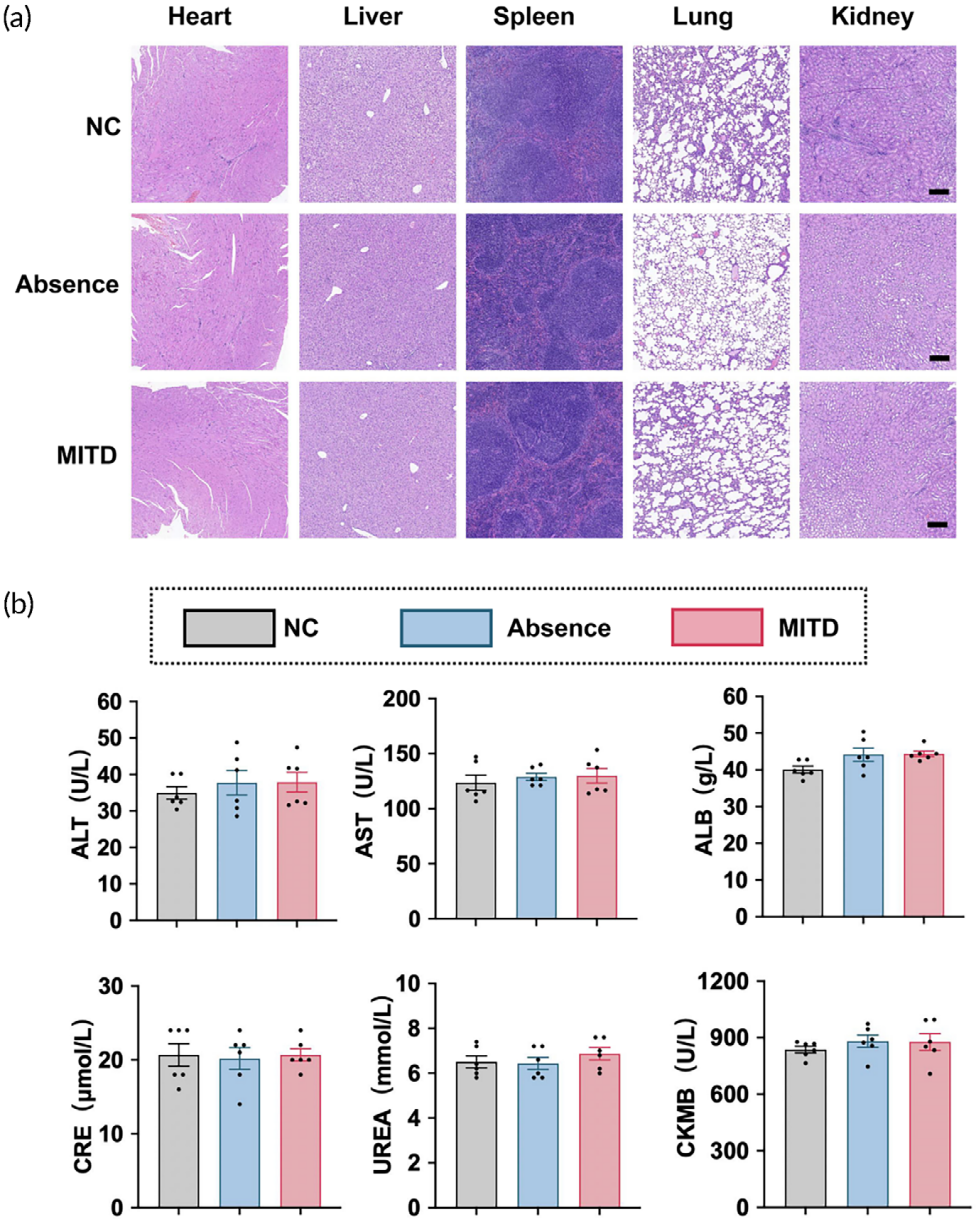
Safety evaluation of MITD‐enhanced RBD mRNA vaccines. (a) H&E staining (scale bar = 200 μm) and (b) analysis of serum biochemical indices following intramuscular administration of 5 μg of RBD mRNA vaccines in BALB/c mice on day 84 (*n* = 6). The groups compared include a normal control (NC), mRNAs containing the MITD (MITD), and mRNAs in the absence of MITD (Absence). The data are shown as mean ± SEM.

It is imperative to emphasize that both the efficacy and safety of vaccines are indispensable. As illustrated in Figure 6a, there were no significant histopathological differences observed in the heart, liver, spleen, lung, and kidney tissues between the NC, Absence and MITD groups. Biochemical markers, including ALT and AST for liver function, ALB, CRE and UREA for kidney function, and CKMB for heart function, also did not exhibit substantial changes across these groups (Figure 6b). In summary, mRNA vaccines incorporating MITD demonstrated relative safety in these regards.

## 4 DISCUSSION

mRNA vaccines have demonstrated a significant and potent role in combating infectious diseases and tumors.^35^ To date, a variety of strategies have been proposed to enhance the efficacy of stable and promising LNPs‐based mRNA vaccines. These strategies primarily involve the development of novel lipid carrier materials,^24^ incorporation of adjuvants,^26^ addition of sequences for self‐assembling protein polymers,^36^, ^37^ optimization of novel 5′ and 3′ UTR sequences,^38^ application of self‐amplifying RNAs (saRNAs),^39^, ^40^ and utilization of circular RNAs (circRNAs).^41^ In establishing our mRNA vaccine system against SARS‐CoV‐2, the notable ionizable lipid material MIC1, along with other components, was selected to encapsulate mRNAs encoding antigens. Comparative analysis revealed that CDS of mRNAs containing MITD exhibited equivalent antigen expression to those lacking MITD in RBD^WT^ mRNA vaccines in vitro. Subsequent in vivo studies indicated that MITD‐enhanced vaccines stimulated higher binding and neutralizing antibody titers against the virus compared to mRNA sequences without MITD. This increased efficacy may be attributed to MITD's enhanced stimulation of antigen‐specific memory B cell responses and a Th1/Tc1 biased memory T cell response. Furthermore, utilizing recent viral mutants as antigens, we observed that MITD‐based mRNA vaccines maintained temporal protection stability, good storage stability, and broad‐spectrum efficacy. Lastly, our study demonstrates that MITD‐based RBD mRNA vaccines exhibit favorable safety profiles.

Interestingly, our research revealed that mRNA vaccines incorporating a classical signal peptide exhibited nearly identical antigen expression levels in vitro to those with an MHCI signal peptide augmented by MITD, at equivalent dosages. However, the MITD group demonstrated an enhanced capacity to stimulate antigen‐specific immune responses. This phenomenon may be attributed to MITD's ability to mimic the dynamic natural transport characteristics of MHC molecules in both immature and mature DCs.^18^ Furthermore, the incorporation of MHCI signal peptide with MITD was found to stimulate both CD4^+^ and CD8^+^ cells, extending beyond the CD8^+^ T cells typically associated with MHCI. This cross‐presentation effect is likely due to the MITD trafficking signal, aligning with previous findings.^18^, ^20^, ^21^ To the best of our knowledge, in the context of infectious disease treatment, MITD has been tailored for SARS‐CoV‐2 and *M. tuberculosis* mRNA vaccines through computational simulations, yet there has been a lack of comprehensive in vivo comparison of efficacy and immune mechanisms, as well as a basis for selection.^42^, ^43^ Beyond this approach of targeting organelles, researchers have also explored genetic modifications involving lysosome‐associated membrane protein (LAMP),^44^, ^45^, ^46^ MHCII invariant chain fusion proteins,^47^, ^48^ and H2‐M/O^49^ to effectively route antigens to endosomal processing compartments, thereby enhancing major MHC I and/or II presentation. These findings suggest that occasionally integrating other trafficking and localizing signals into antigen sequences could amplify the immune response elicited by DNA or RNA vaccines.

In the germinal center (GC) response, a special subset of differentiated CD4^+^ T cells, namely follicular helper T (Tfh) cells, is required to assist B cells in producing high affinity antibodies against antigen. Tfh cells control the initiation and outcome of germinal center B (GCB) cell responses, which is crucial for the correct production of protective antibodies during infection.^50^, ^51^, ^52^, ^53^ Significantly, although the better activation of MITD‐based mRNA vaccines on Tfh‐GCB axis was not tested in our study, our antigen specific B cell and antibody testing data could already explain the issue (Figures 3 and S1–S3). Furthermore, the Tfh and GCB examination could be taken into account when clarifying the specific mechanism of vaccines.

While several mRNA vaccines undergoing clinical trials have been designed based on the S protein of SARS‐CoV‐2,^54^, ^55^ the RBD of the virus has emerged as a promising vaccine antigen. This is primarily due to the virus's mechanism of entry into alveolar cells for replication, which involves direct interaction between the RBD and the angiotensin‐converting enzyme 2 receptor (ACE2).^56^, ^57^, ^58^ Furthermore, RBD monomers offer the advantages of a limited size and reduced immunogenicity, rendering them less susceptible to detection by the immune system.^36^, ^59^ Additionally, antigens derived from the WT SARS‐CoV‐2 have shown diminished neutralizing and binding antibody titers against variant strains,^60^ possibly due to their ability to evade vaccine‐induced and infection‐induced immunity.^61^, ^62^ In our study, we observed that even for Delta and Omicron variants, which traditionally exhibit relatively poor protection efficiency, the RBD^WT^ antigen linked with an enhanced MITD conferred effective protection. Moreover, the protective efficacy of the enhanced MITD‐based RBD^WT^ antigen against variants was found to be comparable to that of vaccines lacking MITD when tested against the corresponding WT strain (Figures 3c–f and S1–S3).

Although the Omicron variants exhibit reduced replication and pathogenicity, they possess a pronounced ability to evade the neutralizing antibodies elicited by vaccines.^60^ This observation prompted our investigation into the RBD of the recently emerged BQ.1 mutant as the antigen for our subsequent study. Notably, recent SARS‐CoV‐2 Omicron variants, such as XBB.1.5 and CH.1.1, originated from Omicron BA.2, while others like Omicron BF.7 and BQ.1 descended from Omicron BA.5.^34^ This lineage may explain why the BQ.1 antigen demonstrates enhanced preventative efficacy against BQ.1, BF.7, and BA.5 compared to B.1.1.529, XBB.1.5, and CH.1.1 (Figures 5d and S6). Despite the diminished protective capacity of vaccines using the WT SARS‐CoV‐2 antigen against recent variants, our research revealed that the MITD‐enhanced RBD^BQ.1^ mRNA vaccines exhibited superior preventive effects against certain later Omicron variants. Notably, in both the WT and Omicron BQ.1‐based RBD mRNA vaccines, the durability of binding and neutralizing antibodies conferred by MITD was observed to be both high and lasting. This outcome suggests that the MITD design could potentially be universally applicable to other mRNA vaccines targeting RNA virus infectious diseases.

## 5 CONCLUSIONS

In conclusion, the integration of the major histocompatibility complex class I trafficking domain (MITD) into the sequences of mRNA vaccines represents a viable strategy to amplify immune responses. The MITD‐based design demonstrated a superior immunostimulatory effect compared to formulations lacking MITD, even at equivalent levels of antigen expression. Our findings lay a foundational framework for the ongoing development of efficacious mRNA vaccines aimed at treating a range of RNA virus diseases.

## AUTHOR CONTRIBUTIONS

**Yupei Zhang:** Conceptualization; data curation; project administration; software; writing – original draft; writing – review and editing. **Songhui Zhai:** Formal analysis; investigation; writing – review and editing. **Shugang Qin:** Data curation; funding acquisition; resources; software; writing – review and editing. **Yuting Chen:** Software; validation; visualization; writing – original draft; writing – review and editing. **Kepan Chen:** Methodology; resources. **Zhiying Huang:** Project administration. **Xing Lan:** Project administration. **Yaoyao Luo:** Methodology. **Guohong Li:** Project administration. **Hao Li:** Project administration. **Xi He:** Resources. **Meiwan Chen:** Funding acquisition. **Zhongwei Zhang:** Funding acquisition; resources. **Xingchen Peng:** Funding acquisition; supervision; validation. **Xin Jiang:** Conceptualization; funding acquisition; resources. **Hai Huang:** Conceptualization; formal analysis; funding acquisition; methodology; supervision; writing – review and editing. **Xiangrong Song:** Conceptualization; funding acquisition; resources; supervision; validation; writing – review and editing.

## CONFLICT OF INTEREST STATEMENT

The authors have no conflicts of interest to declare.

## Supporting information


**FIGURE S1.** Serum ELISA of (a) RBD^WT^ and (b) RBD^Delta^‐specific IgG titers at day 14 in BALB/c mice vaccinated with 0.2, 1, and 5 μg RBD^WT^ mRNA vaccines of MITD and Absence (related to Figure 3c,d). The groups compared include a normal control (NC), mRNAs containing the MITD (MITD), and mRNAs in the absence of MITD (Absence). The data are shown as mean ± SEM, *n* = 4. **p < 0.05, ***p < 0.01.
**FIGURE S2.** Serum ELISA of RBD^Omicron^‐specific IgG titers at day 14 in BALB/c mice vaccinated with 5 μg RBD^WT^ mRNA vaccines of MITD and Absence (related to Figure 3e). The groups compared include a normal control (NC), mRNAs containing the MITD (MITD), and mRNAs in the absence of MITD (Absence). The data are shown as mean ± SEM, *n* = 4. **p < 0.05.
**FIGURE S3.** Serum ELISA of WT, Delta, and Omicron B.1.1.529 RBD‐specific IgG titers at day (a) 42 and (b) 84 in BALB/c mice vaccinated with 5 μg MITD‐based RBD^WT^ mRNA vaccines of MITD and Absence (related to Figure 3c‐e). The groups compared include a normal control (NC), mRNAs containing the MITD (MITD), and mRNAs in the absence of MITD (Absence). The data are shown as mean ± SEM, *n* = 4. **p < 0.05, ***p < 0.01.
**FIGURE S4.** B cell gating strategy (related to Figure 3g,h). Cells were gated as singlets and live cells on forward and side scatter and a live/dead stain. CD3^−^, CD4^−^ cells were then gated on absence of CD14 and CD16 expression and positive expression of CD20 and CD19. Memory B cells were selected based on lack of IgD or IgM. Finally, S protein probes were used to determine binding specificity.
**FIGURE S5.** T cell gating strategy (related to Figure 4a,b). Cells were gated as singlets and live cells on forward and side scatter and a live/dead stain. CD3^+^ and CD44^+^ cells were selected on MHCII^−^ and B220^−^ cells. Th cells were gated by CD4^+^, and cytotoxic T cells were gated by CD8^+^. Finally, ICS of IFN‐γ^+^, TNF‐α^+^, and IL‐2^+^ cells in CD4^+^ and CD8^+^ cells, respectively, were used to determine memory T cell response.
**FIGURE S6.** Pseudovirus neutralization test of MITD‐based RBD^BQ.1^ mRNA vaccines on (a) Omicron B.1.1.529, (b) XBB.1.5, and (c) CH.1.1 variant of SARS‐CoV‐2 (related to Figure 5d). The data are shown as mean ± SEM, *n* = 6. ***p < 0.01.

## Data Availability

Data available on request from the authors.
