# Supplementary material for: MHCI trafficking signal‐based mRNA vaccines strengthening immune protection against RNA viruses
Source: Bioeng Transl Med. 2024 Aug 15;10(1):e10709. doi: 10.1002/btm2.10709 (PMC11711215; doi:10.1002/btm2.10709)
Supplement: Supplementary file 1 — FIGURE S1. Serum ELISA of (a) RBDWT and (b) RBDDelta‐specific IgG titers at day 14 in BALB/c mice vaccinated with 0.2, 1, and 5 μg RBDWT mRNA vaccines of MITD and Absence (related to Figure 3c,d). The groups compared include a normal control (NC), mRNAs containing the MITD (MITD), and mRNAs in the absence of MITD (Absence). The data are shown as mean ± SEM, n = 4. **p < 0.05, ***p < 0.01. FIGURE S2. Serum ELISA of RBDOmicron‐specific IgG titers at day 14 in BALB/c mice vaccinated with 5 μg RBDWT mRNA vaccines of MITD and Absence (related to Figure 3e). The groups compared include a normal control (NC), mRNAs containing the MITD (MITD), and mRNAs in the absence of MITD (Absence). The data are shown as mean ± SEM, n = 4. **p < 0.05. FIGURE S3. Serum ELISA of WT, Delta, and Omicron B.1.1.529 RBD‐specific IgG titers at day (a) 42 and (b) 84 in BALB/c mice vaccinated with 5 μg MITD‐based RBDWT mRNA vaccines of MITD and Absence (related to Figure 3c‐e). The groups compared include a normal control (NC), mRNAs containing the MITD (MITD), and mRNAs in the absence of MITD (Absence). The data are shown as mean ± SEM, n = 4. **p < 0.05, ***p < 0.01. FIGURE S4. B cell gating strategy (related to Figure 3g,h). Cells were gated as singlets and live cells on forward and side scatter and a live/dead stain. CD3−, CD4− cells were then gated on absence of CD14 and CD16 expression and positive expression of CD20 and CD19. Memory B cells were selected based on lack of IgD or IgM. Finally, S protein probes were used to determine binding specificity. FIGURE S5. T cell gating strategy (related to Figure 4a,b). Cells were gated as singlets and live cells on forward and side scatter and a live/dead stain. CD3+ and CD44+ cells were selected on MHCII− and B220− cells. Th cells were gated by CD4+, and cytotoxic T cells were gated by CD8+. Finally, ICS of IFN‐γ+, TNF‐α+, and IL‐2+ cells in CD4+ and CD8+ cells, respectively, were used to determine memory T cell response. FIGURE S6. Pseudovirus ne [file BTM2-10-e10709-s001.docx]

# **Supplementary Figures**

**
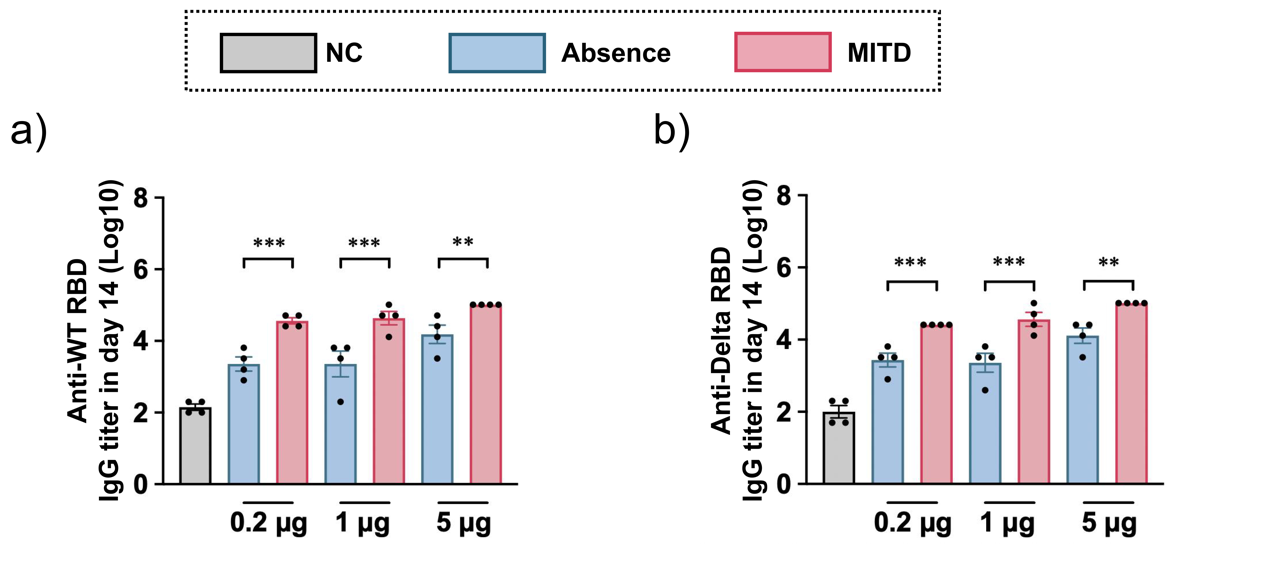
Figure S1.** Serum ELISA of (a) RBD^WT^ and (b) RBD^Delta^-specific IgG titers at day 14 in BALB/c mice vaccinated with 0.2, 1 and 5 μg RBD^WT^ mRNA vaccines of MITD and Absence (related to Figure 3c,d). The groups compared include a normal control (NC), mRNAs containing the MITD (MITD), and mRNAs in the absence of MITD (Absence). The data are shown as mean ± SEM, n = 4. ^**^*P* < 0.05, ^***^*P* < 0.01.

**
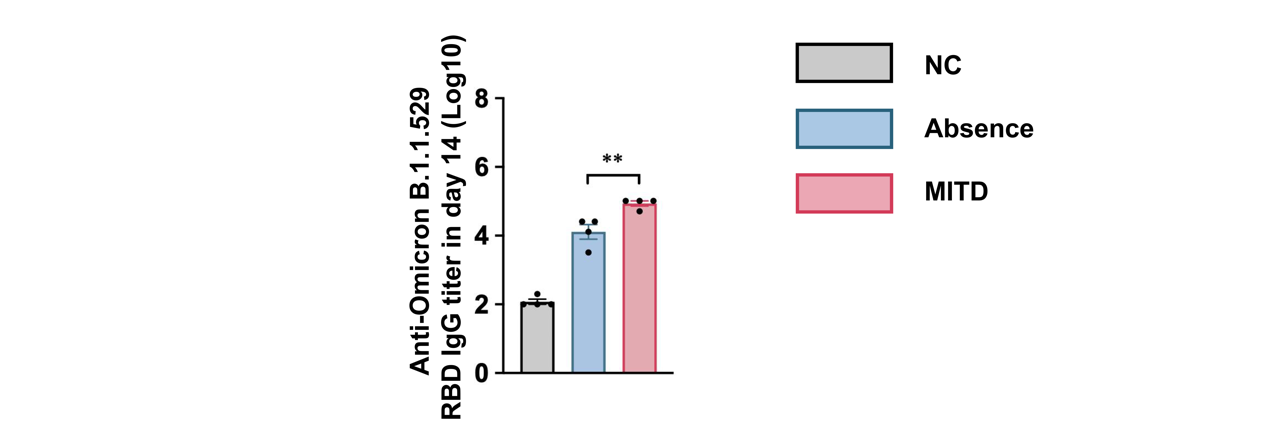
Figure S2.** Serum ELISA of RBD^Omicron^-specific IgG titers at day 14 in BALB/c mice vaccinated with 5 μg RBD^WT^ mRNA vaccines of MITD and Absence (related to Figure 3e). The groups compared include a normal control (NC), mRNAs containing the MITD (MITD), and mRNAs in the absence of MITD (Absence). The data are shown as mean ± SEM, n = 4. ^**^*P* < 0.05.

**
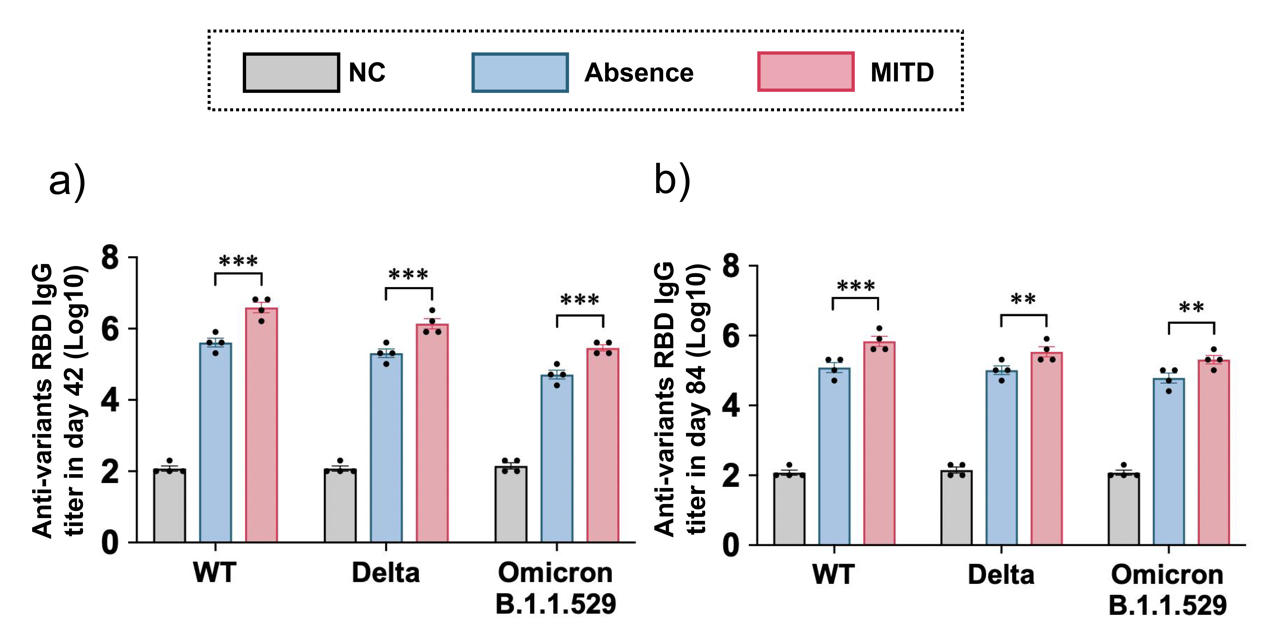
Figure S3.** Serum ELISA of WT, Delta and Omicron B.1.1.529 RBD-specific IgG titers at day (a) 42 and (b) 84 in BALB/c mice vaccinated with 5 μg MITD-based RBD^WT^ mRNA vaccines of MITD and Absence (related to Figure 3c-e). The groups compared include a normal control (NC), mRNAs containing the MITD (MITD), and mRNAs in the absence of MITD (Absence). The data are shown as mean ± SEM, n = 4. ^**^*P* < 0.05, ^***^*P* < 0.01.

**
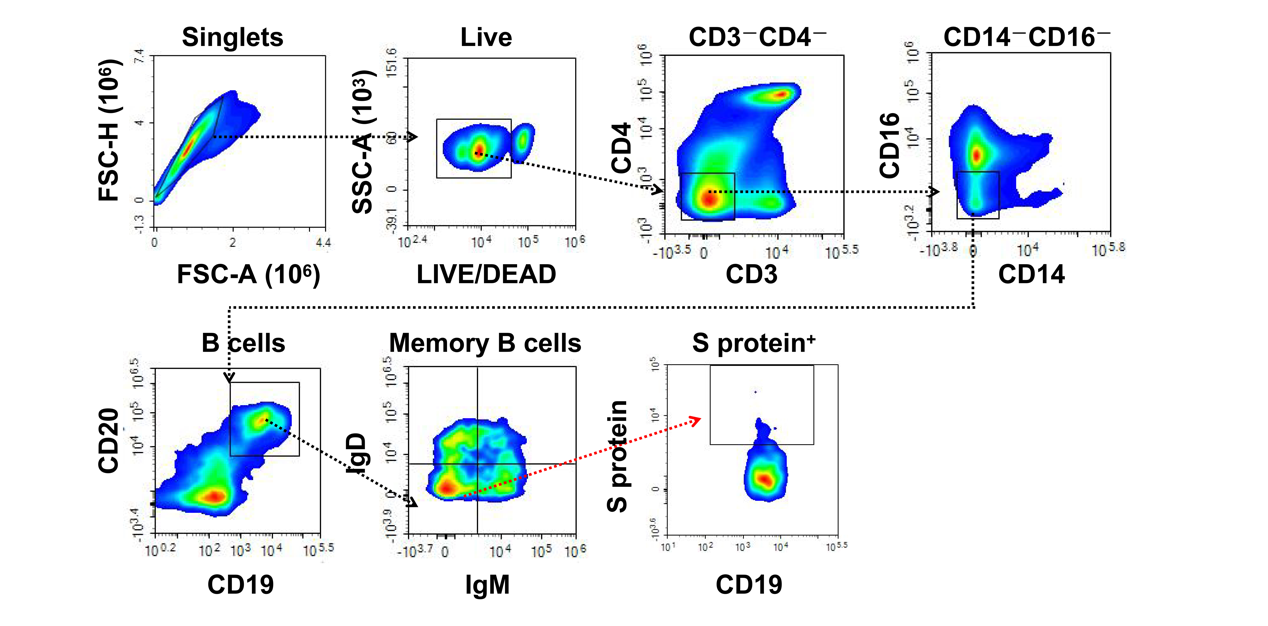
Figure S4.** B cell gating strategy (related to Figure 3g, h). Cells were gated as singlets and live cells on forward and side scatter and a live/dead stain. CD3^－^, CD4^－^ cells were then gated on absence of CD14 and CD16 expression and positive expression of CD20 and CD19. Memory B cells were selected based on lack of IgD or IgM. Finally, S protein probes were used to determine binding specificity.

**
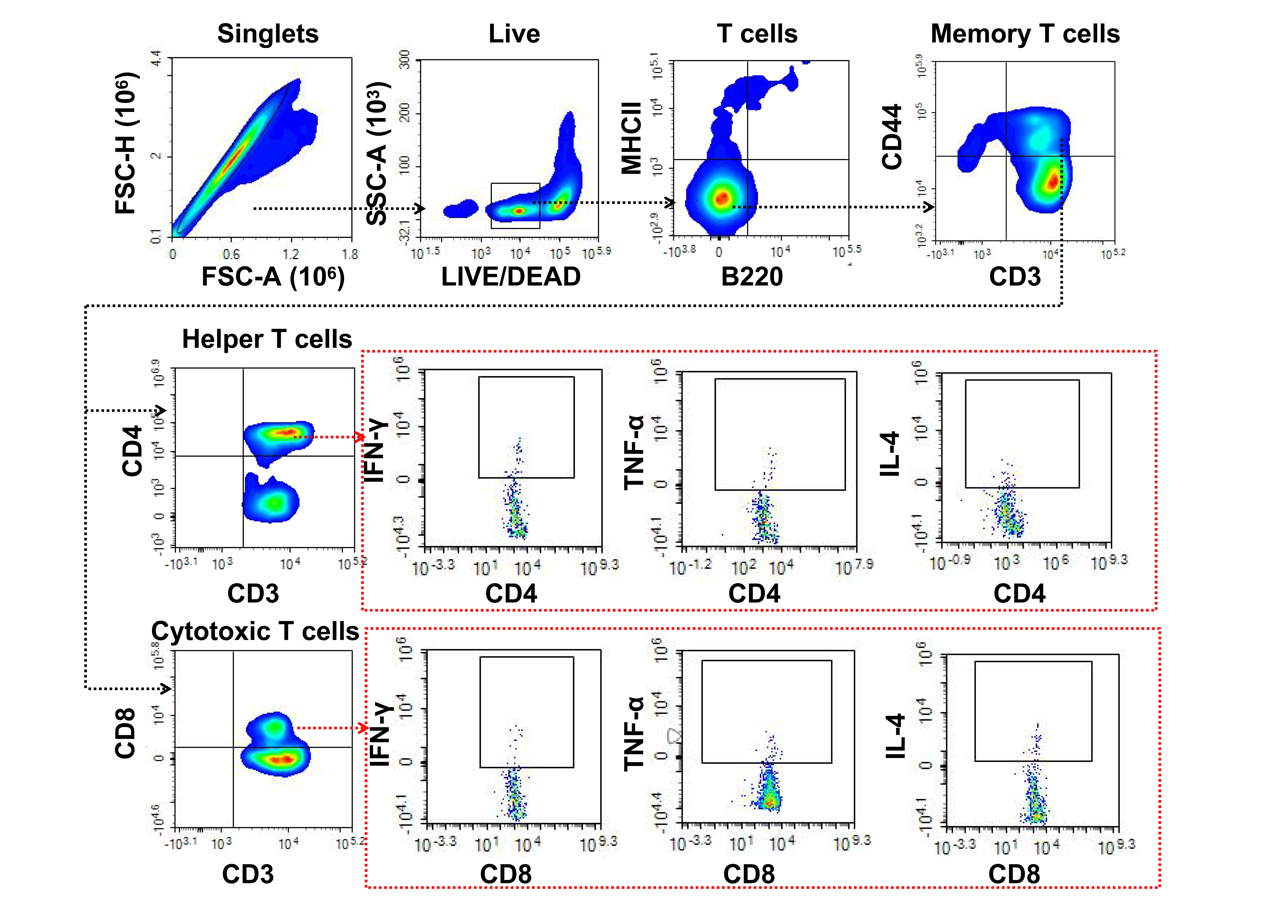
Figure S5.** T cell gating strategy (related to Figure 4a, b). Cells were gated as singlets and live cells on forward and side scatter and a live/dead stain. CD3^+^ and CD44^+^ cells were selected on MHCII^－^ and B220^－^ cells. Th cells were gated by CD4^+^, and cytotoxic T cells were gated by CD8^+^. Finally, ICS of IFN-γ^+^, TNF-α^+^and IL-2^+^ cells in CD4^+^ and CD8^+^ cells, respectively, were used to determine memory T cell response.


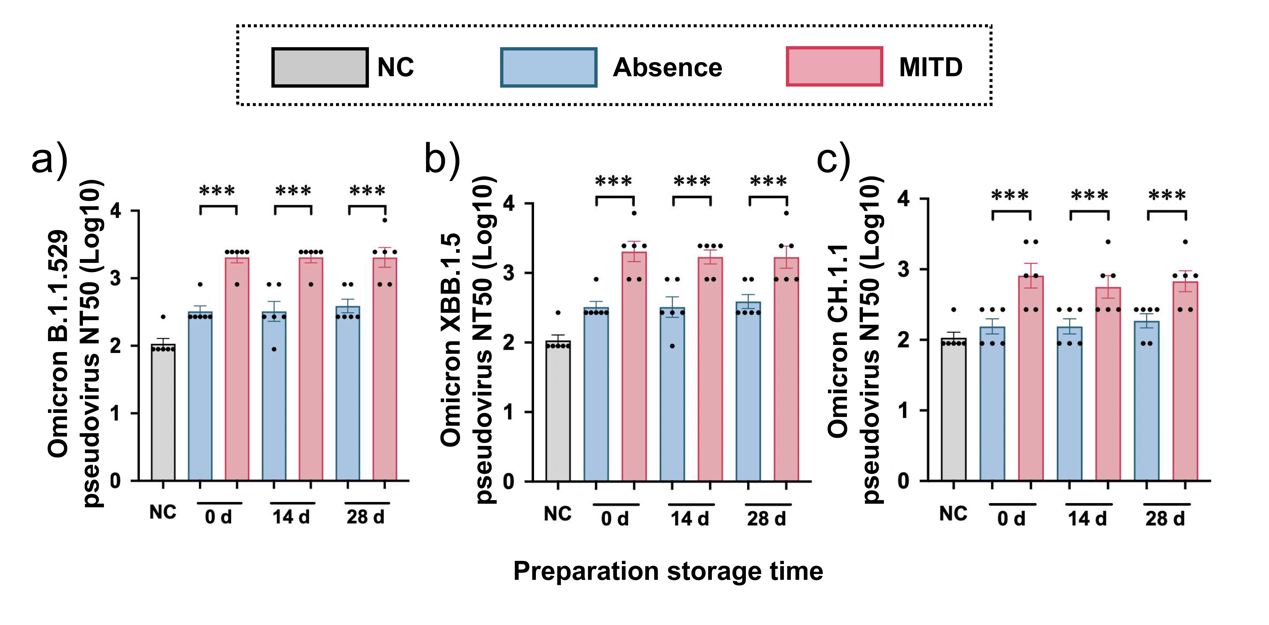


**Figure S6.** Pseudovirus neutralization test of MITD-based RBD^BQ.1^ mRNA vaccines on (a) Omicron B.1.1.529, (b) XBB.1.5 and (c) CH.1.1 variant of SARS-CoV-2 (related to Figure 5D). The data are shown as mean ± SEM, n = 6. ^***^*P* < 0.01.
